# MassARRAY: a high-throughput solution for rapid detection of foodborne pathogens in real-world settings

**DOI:** 10.3389/fmicb.2024.1403579

**Published:** 2024-06-25

**Authors:** Namfon Suebwongsa, Surasak Jiemsup, Pannita Santiyanont, Piyapha Hirunpatrawong, Pornsiri Aswapairin, Monthathip Thongkum, Prakaymars Panumars, Nipa Chokesajjawatee, Supaporn Wongsrichai, Pichet Koompa, Suganya Yongkiettrakul

**Affiliations:** ^1^National Center for Genetic Engineering and Biotechnology (BIOTEC), National Science and Technology Development Agency (NSTDA), Pathum Thani, Thailand; ^2^Lifomics Co., Ltd., Bangkok, Thailand; ^3^Bureau of Quality Control of Livestock Products, Department of Livestock Development, Ministry of Agriculture and Cooperatives, Pathum Thani, Thailand

**Keywords:** foodborne pathogenic bacteria, food safety, MassARRAY, mass spectrometer, high-throughput genotyping

## Abstract

**Introduction:**

Bacterial foodborne pathogens pose a substantial global public health concern, prompting government agencies and public health organizations to establish food safety guidelines and regulations aimed at mitigating the risk of foodborne illness. The advent of DNA-based amplification coupled with mass spectrometry, known as MassARRAY analysis, has proven to be a highly precise, sensitive, high-throughput, and cost-effective method for bacterial detection. This study aimed to develop, validate, and evaluate a MassARRAY-based assay for the detection and identification of significant enteropathogenic bacteria.

**Methods:**

The MassARRAY-based assay was developed for the detection of 10 crucial bacterial foodborne pathogens, including *Campylobacter coli*, *Campylobacter jejuni*, *Clostridium perfringens*, *Escherichia coli*, *Enterococcus faecalis*, *Enterococcus faecium*, *Listeria monocytogenes*, *Salmonella* spp., *Shigella* spp., and *Staphylococcus aureus*. The assay was optimized using the reference gDNA (*n* = 19), followed by validation using gDNA (*n* = 85) of reference and laboratory isolates. Additionally, the evaluation of the assay’s reaction using a mixture of gDNA from all nine targeted species was performed. The limit of detection of the developed MassARRAY-based assay was determined using bacterial cells. Moreover, the validation method for field samples was evaluated by comparing it with standard microbiological testing methods routinely analyzed.

**Results:**

The developed MassARRAY-based assay demonstrated 100% concordance with known bacterial pure cultures. The assay’s reaction using a mixture of gDNA from all nine targeted species revealed the MassARRAY’s capability to detect all targeted species in a single assay with the lowest concentration of 1 ng/μL of gDNA. The limits of detection of the assay range from 357 ± 101 to 282,000 ± 79,196 cells. Moreover, the validation of the assay in field samples revealed a 100% correlation between the data obtained from the standard microbiological method and the MassARRAY-based assay.

**Discussion:**

These findings suggested that the developed MassARRAY-based assay exhibited the excellence in high-throughput detection of foodborne bacterial pathogens with high accuracy, reliability, and potential applicability within real-world field samples.

## 1 Introduction

Foodborne illnesses exert a pervasive global impact, affecting populations worldwide. The World Health Organization (WHO) has estimated that 600 million people suffer from foodborne illnesses annually, resulting in 420,000 deaths and unsafe food consumption leads to the loss of 33 million years of healthy lives annually (World Health Organization, 2022). According to the 2019 World Bank report, foodborne diseases impose a substantial economic burden on low- and middle-income countries (LMICs), with an estimated annual cost of approximately $95.2 billion, encompassing productivity losses, while the annual cost of treating foodborne illnesses is estimated at $15 billion (Jaffee et al., 2019). These findings underscore the significant financial impact of foodborne illnesses, highlighting the urgent need for effective prevention and control measures.

The imperative for efficient technology to detect foodborne pathogens stems from the substantial impact of foodborne diseases. Timely and accurate detection is essential for identifying and mitigating potential outbreaks, improving food safety measures, and reducing the incidence of foodborne illness. Therefore, the development and adoption of advanced technology for rapid pathogen detection are paramount.

Currently, numerous methods are available for pathogen detection (Law et al., 2015; Garzarelli et al., 2022; Tan et al., 2022; Kabiraz et al., 2023). The conventional culture-based method, while widely used, is time-consuming, labor-intensive, and costly, particularly for fastidious bacteria with strict nutritional requirements. To overcome these drawbacks, fast, reliable, cost-effective and high throughput detection of pathogen contamination in food is necessary.

MassARRAY analysis stands as a robust and adaptable technology extensively employed in the realm of molecular biology and genetics research for high-throughput genotyping and mutation detection. The principle of MassARRAY technology involves the simultaneous amplification of multiple targeted DNA sequences in a single reaction, generating amplicons. These amplicons then serve as templates for a single nucleotide extension reaction, and the resulting products with specific molecular masses are subsequently analyzed using Matrix-Assisted Laser Desorption/Ionization Time-Of-Flight Mass Spectrometry (MALDI-TOF MS) (Gabriel et al., 2009). This unique feature enables the identification and analysis of targeted genes based on their molecular masses, eliminating the need for a reference database. This technology presents numerous advantages over alternative genotyping platforms. Its exceptional flexibility and scalability enable the analysis of a few or hundreds of genetic markers in a single experiment.

Over the past decade, MassARRAY has proven its potency in molecular research. It excels in accurately detecting subtle genetic variations, such as single nucleotide polymorphisms (SNPs) (AlMutawa et al., 2023; Wacharapluesadee et al., 2023), and has demonstrated efficiency in cancer mutation detection (Min et al., 2016; Wang et al., 2019), particularly in identifying epidermal growth factor receptor (EGFR) mutations in lung tissue and cytological samples with high sensitivity and specificity, compared to other methods (Min et al., 2016). Furthermore, MassARRAY exhibits robustness in pathogen detection (Peng et al., 2013; Hernandez et al., 2021), notably for detecting specific human enterovirus strains during a hand foot and mouth disease outbreak in China (Peng et al., 2013). It has also been implemented in detecting antimicrobial resistant genes (Syrmis et al., 2011; Zowawi et al., 2018), showing superior sensitivity and specificity, compared to conventional methods (Syrmis et al., 2011). Recently, MassARRAY has emerged as a reliable method for detecting and genotyping pathogens in COVID-19 infection samples (Hernandez et al., 2021), even proving its effectiveness in identifying and subtyping various SARS-CoV-2 variants (Wacharapluesadee et al., 2023). These achievements highlighted the significant potential of MassARRAY as a valuable tool, showing promising prospects for its application in a wide range of pathogen detection and genotyping.

This study aimed to develop, validate, and evaluate a MassARRAY-based assay for the detection and identification of significant enteropathogenic bacteria, including *Campylobacter coli*, *C. jejuni*, *Clostridium perfringens*, *Escherichia coli*, *Shigella* spp., *Enterococcus faecalis*, *E. faecium*, *Listeria monocytogenes*, *Salmonella* spp., and *Staphylococcus aureus*. By prioritizing these pivotal enteropathogenic bacteria, the study attempted to leverage the potential of MassARRAY for accurate and efficient detection and identification. This contributes to an enhancement of diagnostic capabilities in the field of foodborne bacteria.

## 2 Materials and methods

### 2.1 Reference genomic DNA samples and bacterial strains

Reference genomic DNA (gDNA) samples (*n* = 19; Supplementary Table S1) including 13 strains of targeted bacteria (*Campylobacter coli*, *C. jejuni*, *Clostridium perfringens*, *Escherichia coli*, *Enterococcus faecalis* (2 strains), *Enterococcus faecium*, *L. monocytogenes* (2 strains), *Salmonella enterica* (2 strains), *Shigella* spp., and *Staphylococcus aureus*), non-targeted bacteria (*Listeria marthii*), protozoa (*Crithidia fasciculata* and *Leishmania martiniquensis*), severe acute respiratory syndrome coronavirus 2 (SARS-CoV-2), human (Applied Biosystems, California, United States), and plant (teak leave), were utilized as DNA templates for developing and optimizing the MassARRAY-based assay. A collection of 85 laboratory bacterial strains, sourced from diverse environments, animals, and humans (Supplementary Table S2) were used for assay evaluations. These bacterial strains had undergone rigorous species identification, using various methods, including conventional bacterial culture with biochemical tests, conventional PCR, and whole-genome sequencing.

### 2.2 Bacterial culture conditions

*Escherichia coli*, *Shigella* spp., *Klebsiella* spp., *Listeria* spp., *Salmonella* spp., and *S. aureus* were cultured on tryptic soy agar (Difco Laboratories Inc., Franklin Lakes, New Jersy, United States) at 37°C for 18–24 h. *Enterococcus* spp. was grown on De Man–Rogosa–Sharpe (MRS) (Difco Laboratories Inc., Franklin Lakes, New Jersy, United States) at 37°C for 24–48 h. *Campylobacter* spp. was grown on sheep blood agar (Thermo Fisher Scientific Inc. Massachusetts, United States) at 42°C for 24 h under micro-aerophilic condition using AnaeroPack™-MicroAero (Mitsubishi Gas Chemical Company, Inc., Japan). *Streptococcus* spp. was grown on Columbia blood agar (Thermo Fisher Scientific Inc. Massachusetts, United States) at 37°C, in 5% CO_2_ incubator for 18–24 h. *Vibrio parahaemolyticus* was cultured overnight on tryptic soy agar (TSA, Difco Laboratories Inc., Franklin Lakes, New Jersy, United States) with 2% NaCl at 37°C in 5% CO_2_ incubator for 16–18 h. *Clostridium* spp. was grown on Reinforced Clostridial Medium (RCM) (Difco Laboratories Inc., Franklin Lakes, New Jersy, United States) under anaerobic condition (10% H_2_/10% CO_2_/80% N_2_) at 37°C for 48 h. Following the culturing process, a single colony of each bacterial species was selected for the bacterial culture to prepare gDNA subsequently.

### 2.3 Genomic DNA preparation

Three distinct DNA extraction kits were employed to ensure comprehensive and reliable DNA isolation for specific purposes in this study. The ZymoBIOMICS DNA Miniprep Kit (Zymo Research Corp, California, United States) was used for gDNA extraction following the manufacturer’s protocol, yielding highly pure genomic DNA suitable for primer specificity testing and method development experiments. For MassARRAY-based bacterial detection in field samples, the MagPurix^®^ Bacterial DNA Extraction Kit (ZP02006) (Zinexts Life Science Crop., New Taipei, Taiwan) in combined with MagPurix^®^ 12EVO automatic instruments was utilized to obtain high-quality gDNA templates. Heat-lysis extraction using QuickExtract DNA Extraction Solution (LGC Biosearch Technologies, Hoddesdon, United Kingdom) was conducted to obtain gDNA samples for the LOD determination experiment. The quality of extracted gDNA was evaluated using a NanoDrop spectrophotometer (Thermo Fisher Scientific Inc., Massachusetts, United States). Samples with an OD260/280 ratio ranging from 1.8 to 2.0 and an OD260/230 ratio within the range of 2.0 to 2.2 were chosen for subsequent experiments.

### 2.4 Preparation of field samples for MassARRAY-based assay

Meat samples from various sources (*n* = 103) preserved in culture transport medium, along with bacterial culture plates, including both selective and enrichment plates, were generously provided by the Bureau of Quality Control of Livestock Products (BQCLP), Department of Livestock Development, Ministry of Agriculture and Cooperatives, Thailand. The preparation of meat samples and bacterial cell culture was conducted at the BQCLP laboratory (ISO/IEC 17025), adhering to FDA’s Bacteriological Analytical Manual (BAM) guidelines, including those for aerobic plate count (FDA’s BAM 2001, Chapter 3), *Coliform* and *E. coli* (FDA’s BAM 2020 Chapter 4), and *Clostridium* spp. (FDA’s BAM 2001, Chapter 16 and API). Additionally, protocols outlined in the International Organization for Standardization (ISO) Food Production Guideline (ISO 6759:2017/Amd. 1:2020 for *Salmonella* spp. and ISO 6888-3:2003 for *S. aureus*), and Thailand’s Integrated Antimicrobial Resistance Surveillance with One Health Approach Guideline were followed (National Antimicrobial Resistant Surveillance Center, Thailand, 2023; The United States Food and Drug Administration, 2024). Briefly, 25–50 g of meat samples were transferred to culture transport mediums, such as DF (0.1% Peptone normal saline solution), BPB (Butterfield’s Phosphate-Buffered), and BPW (Buffered Peptone Water), in accordance with standard protocols. Subsequently, approximately 5 mL of culture samples were processed for gDNA extraction. For bacterial culture plate samples, a few colonies were selected for gDNA extraction. The DNA extraction procedure employed the MagPurix^®^ Bacterial DNA Extraction Kit (ZP02006), following the manufacturer’s instructions. The high-quality gDNA samples obtained were subsequently used as DNA templates for bacterial identification using the developed MassARRAY-based assay.

### 2.5 Primer and assay design

Species-specific genes were chosen based on primer sequences provided in previously reported PCR-based methods (Liu, 2011). The primer design process involved retrieving a substantial number of full coding sequences from the NCBI genome database. For each targeted gene, a substantial number of sequences, ranging from approximately 100–2,000 sequences, were obtained. These sequences were then subjected to multiple sequence alignment using an alignment tool, CLUSTAL-W (Thompson et al., 1994) to identify highly conserved regions within the sequences across different strains or isolates of the same species. The highly conserved regions of the species-specific targeted genes were subsequently employed for primer design using Assay Design 4.0 software (Agena Bioscience, Inc., California, United States).

All targeted sequences were inputted into Assay Design 4.0 software (Agena Bioscience, Inc., California, United States) for primer and assay design, following the manufacturer’s recommendations. The primer design process adhered to stringent parameters to minimize primer-dimer formation, hairpin loops, and false priming, ensuring the production of high-quality and specific primers. A set of primers specific for the conserved region of the *16S rRNA* gene was included as an internal control, enabling the detection of any species of bacteria, if presence in the sample. The specificity of designed primers was evaluated using the Basic Local Alignment Search Tool (BLAST) to ensure the highest level of primer specificity was achieved. Subsequently, each primer pair (forward and reverse primers) and different set of multiplex primers were rigorously evaluated using conventional PCR reaction with gDNA samples from targeted bacteria, non-targeted bacteria, and other organisms, as well as negative control (no DNA template) to ensure primer specificity and absence of cross-PCR reactions. Only primers exhibiting the highest specificity were selected for use in the MassARRAY-based assay system. It is important to highlight that manual adjustments, such as altering bases and adjusting primer length, were implemented as necessary to achieve uniformity of melting temperature (Tm) and guanine-cytosine content (%GC) values among multiplex primers, facilitating PCR optimization. All primers were synthesized by Macrogen, Inc. (Macrogen, Inc., Seoul, Korea).

### 2.6 Optimization of MassARRAY protocol and analysis

The initial step of the MassARRAY-based assay involved optimizing a multiplex PCR reaction, using gDNA of known bacterial strains as reference DNA templates (*n* = 14; Supplementary Table S1). *L. marthii* (non-targeted bacterium) and the non-bacterial gDNA samples, including *C. fasciculata* and *L. martiniquensis*, SARS-CoV-2, human, and plant, were used as negative controls. The MassARRAY-based assay panel was optimized and modified based on the manufacturer’s recommended protocol. In brief, the 5 μL of PCR reaction consisted of 2.5 μL of EconoTaq^®^ PLUS 2X Master Mix (LGC Biosearch Technologies, Hoddesdon, United Kingdom), 0.5 μL of the primer mixture (resulting in a final concentration of 500 nM for each forward and reverse primer), 1.0 μL of gDNA template, and 1 μL of DNase-free distilled water. The PCR reaction includes: an initial denaturation at 94°C for 2 min, followed by 35 cycles of denaturation at 94°C for 30 s, annealing at 55°C for 30 s, extension at 72°C for 1 min, and a final extension step at 72°C for 5 min. The reaction was then cooled to 4°C to stop the reaction. Following the PCR reaction, the excess dNTPs in the reaction were eliminated by shrimp alkaline phosphatase (SAP) (Agena Bioscience, California, United States). The dephosphorylation reaction was performed at 37°C for 40 min, and then inactivated at 85°C for 5 min. Following the SAP reaction, the single-base extension (SBE) reaction was conducted using a mixture of extension reaction cocktail that was prepared according to the iPLEX^®^ Pro and Gold Reagents User Guide. The SBE reaction was performed with an initial step at 95°C for 30 s, followed by 40 cycles consisting of denaturation at 95°C for 5 s, and 5 cycles of an annealing step at 52°C for 5 s and a denaturation step at 80°C for 5 s. Finally, the extension was carried out at 72°C for 3 min, and the reaction was then held at 4°C. Subsequently, the SBE products were dispensed onto the SpectroCHIP through a nano-dispenser and then loaded into the mass spectrometer (MS) for the measurement of the molecular mass of the SBE products (Agena Bioscience, California, United States). The molecular mass and base calling data corresponding to specific SBE products acquired from the MS were analyzed and interpreted by MassARRAY^®^ Typer Viewer v4.0 software (Agena Bioscience, California, United States).

### 2.7 Validation and evaluation of MassARRAY-based assay

The developed MassRRAY-based assay was validated using purified gDNA from 85 bacterial strains, including 75 strains of the targeted bacterial species and 10 strains of non-targeted bacterial species (Supplementary Table S2). For each assay reaction, 10 ng/μL of purified gDNA were used as a sample, and the experiments were done independently in triplicate. The correlation between the developed MassARRAY-based assay results and known bacterial isolates was determined.

The efficiency of the developed MassARRAY-based assay’s reaction was evaluated using a mixture of gDNA from all nine targeted species, including *C. coli*, *C. jejuni*, *Cl. perfringens*, *E. coli*/*Shigella* spp., *E. faecalis*, *E. faecium*, *L. monocytogenes* (variant-1), *L. monocytogenes* (variant-2), *Salmonella* spp., and *S. aureus*. The gDNA from each targeted species was combined, with concentrations varying from 10 to 50 ng/μL for each species. Subsequently, 1 μL of the gDNA mixture was utilized as the template, resulting in a final concentration of 1–5 ng/μL for each targeted species. The experiments were conducted independently and in duplicate.

The limit of detection of the developed MassARRAY-based assay was determined using bacterial cells. The bacterial culture was adjusted to an optical density (OD) of 1.85 at 600 nm, equivalent to approximately 10^9^ colony forming units (CFU)/mL. The bacterial cell count was determined through a plate count assay using appropriate culture conditions for the species. The bacterial suspension was 10-fold serially diluted to extinction. One hundred microliters of each bacterial dilution were centrifuged to obtain bacterial cell pellets which were subjected to gDNA extraction using 50 μL of QuickExtract DNA Extraction Solution (LGC Biosearch Technologies, Hoddesdon, United Kingdom), following the manufacturing protocol. Briefly, bacterial cell suspensions were subjected to heat-lysis extraction at 65°C for 6 min, followed by thorough vortexing for 15 s. The mixture solution was then incubated at 98°C for 2 min. Subsequently, 1 μL of the extracted gDNA solution was analyzed using the developed MassARRAY-based assay for the LOD assessment. The results were reported as the average values with their corresponding standard deviations. The experiments were conducted independently, with each experiment performed at least in duplicate.

The developed MassARRAY-based assay for bacterial detection was further validated with field samples (*n* = 103). The results obtained from the developed assay were subsequently compared to those results obtained from standard microbiological testing methods routinely performed by the BQCLP laboratory. This comparison ensured the accuracy and reliability of the developed MassARRAY-based assay in bacterial identification.

## 3 Results

### 3.1 Primer design and multiplex PCR reactions

In this study, a MassARRAY-based assay panel was developed for the identification of 10 enteropathogenic bacterial species. The assay panel utilized eleven targeted amplicons to design 11 pairs of multiplex-PCR primers and 12 extension primers. The assay panel comprised eight single-targeted amplicons, including *C. jejuni* (Camp002), *Cl. perfringens* (Clos001), *E. coli*/*Shigella* spp. (Eco001N), *E. faecalis* (Ent001), *E. faecium* (Ent003), *L. monocytogenes* (Lis001), *Salmonella* spp. (Sal002), and *S. aureus* (Stap001), while double-targeted amplicons were designed for *C. coli* (Camp005 and Camp006). Additionally, one target amplicon was designed for the universal detection of bacteria (Bac16 1–1). As a result, 11 pairs of multiplex-PCR primers were specified for each target. An individual extension primer was designed for each amplicon, except for *L. monocytogenes*, which required two extension primers. Since a novel single nucleotide polymorphism (SNP) was discovered in the targeted gene of *L. monocytogenes* strains isolated from samples in Thailand, two extension primers, Lis001 and LisG, were designed to target both wild type (variant-1) and SNP (variant-2), respectively. Therefore, a total of 12 extension primers were utilized in the SBE reaction (Table 1).

**Table 1:** The information of primers designed.

Targeted species	Primers	PCR primers (forward, reverse)		PCR amplicon size (bp)	Extension primers			Mass of SBE products (Da)
		Tm (°C)	% GC		Tm (°C)	% GC	Mass (Da)	
Bacteria	Bac16 1–1	62.8, 66.9	54.5, 48.4	105	45.8	38.9	5545.60	5816.80
*Campylobacter coli*	Camp005	63.5, 61.9	50.0, 45.2	150	50.0	42.9	6747.40	7034.60
	Camp006	63.6, 59.8	50.0, 43.3	105	49.6	39.1	7511.90	7783.10
*Campylobacter jejuni*	Camp002	64.1, 63.5	50.0, 50.0	108	46.5	27.3	6781.50	7108.50
*Clostridium perfringens*	Clos001	61.7, 63.8	46.7, 50.0	101	46.2	21.7	7078.70	7365.90
*Escherichia coli*/*Shigella* spp.	Eco001N	62.0, 63.9	46.7, 50.0	108	48.6	36.8	5795.80	6043.00
*Enterococcus faecium*	Ent001	61.2, 63.9	46.7, 50.0	99	48.4	28.0	7670.00	7957.20
*Enterococcus faecalis*	Ent003	62.6, 63.9	46.7, 50.0	141	49.3	50.0	5425.50	5752.60
*Listeria monocytogenes*	Lis001	62.5, 61.8	50.0, 46.7	99	45.7	56.2	4955.20	5282.30
	LisG	–	–	99	52.1	62.5	4979.20	5306.30
*Salmonella* spp.	Sal002	63.4, 63.8	50.0, 50.0	106	51.1	42.9	6365.20	6652.40
*Staphylococcus aureus*	Stap001	58.6, 59.3	38.7, 43.3	94	46.0	35.0	6238.10	6565.20

According to Assay Design Suite software V4.0, the multiplex-PCR primer lengths were 30–33 bp with a melting temperature (Tm) range of 58.6–66.9°C, and a GC content percentage varying from 38.7 to 54.5%. These multiplex-PCR primers enabled the generation of PCR products with lengths, ranging from approximately 94–150 bp. According to the species-specific sequence selected, the software designed a set of extension primers (EPs) with a Tm range of 45.7–52.1°C, a GC content percentage varying from 21.7 to 62.5%, and molecular mass ranging from 4955.20 to 7670.00 Da, enabling the generation of non-overlapping SBE products with molecular masses ranging from 5282.30 to 7957.20 Da. Detailed information regarding the designed primers was summarized in Table 1.

### 3.2 Specificity and sensitivity of the developed MassARRAY-based assay

**Figure 1:**
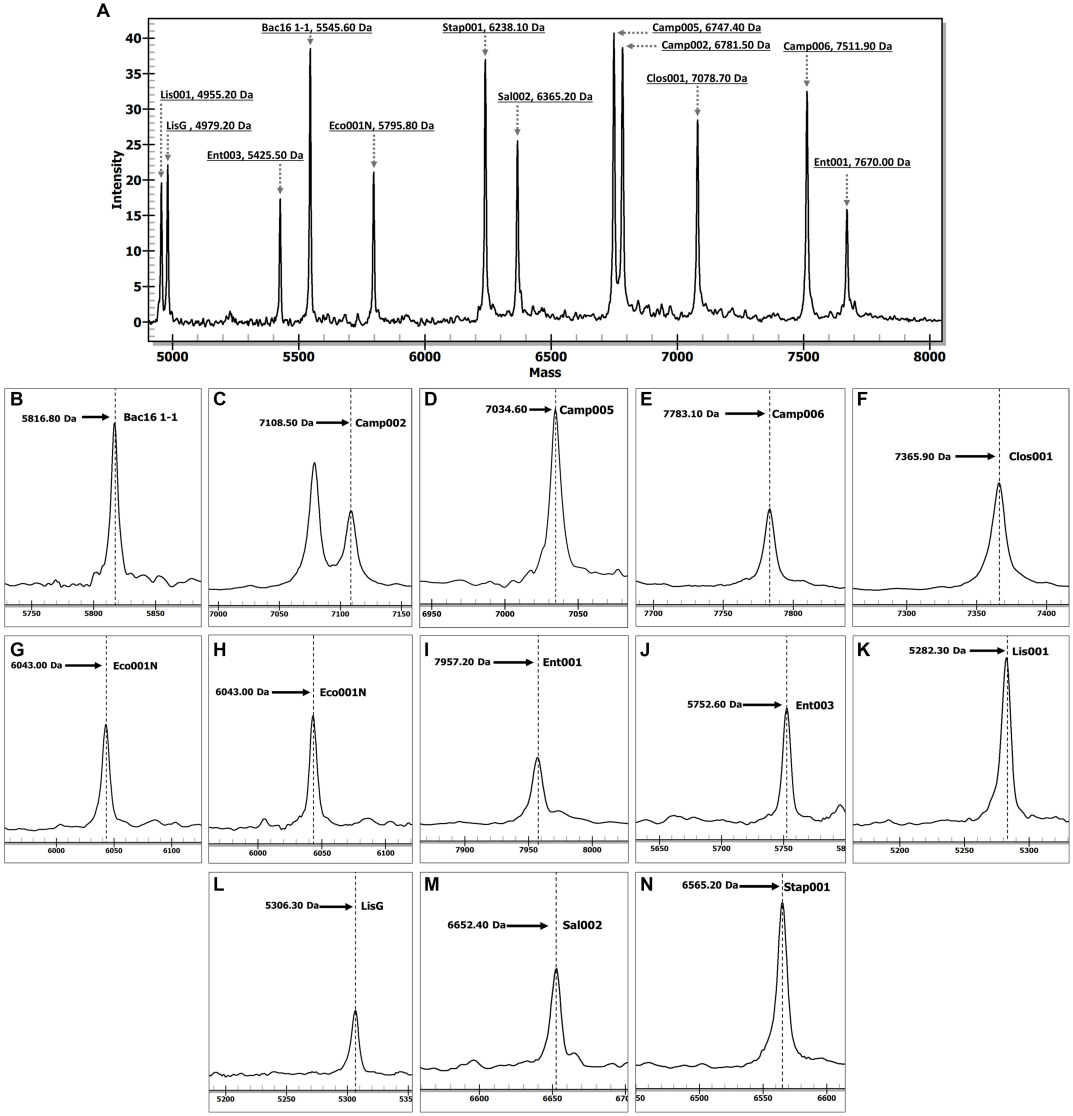
The chromatograms illustrate the specific molecular masses of SBE products corresponding to bacterial targets. Panel **(A)** displays a complete chromatogram illustrating the molecular masses of extension primers in a negative control (DNase-free distilled water) reaction. Panel **(B)** delineates the specific molecular masses of SBE products (Bac16 1–1) for the detection of all bacterial species. Panels **(C)** represent *C. jejuni* SBE specific products while panels **(D,E)** represent *C. coli* SBE specific products. Panels **(F–J)** delineate the specific molecular masses of SBE products for the detection of *Cl. perfringens*, *E. coli, Shigella* spp., *E. faecium*, and *E. faecalis*, respectively. Panels **(K,L)** display the specific molecular masses of SBE products for the detection of *L. monocytogenes* variant-1 and variant-2, respectively, while panels **(M,N)** represent the SBE products for the detection of *Salmonella* spp. and *S. aureus*, respectively. The x-axis represents molecular mass (Da), and the y-axis represents peak intensity.

The MassARRAY-based assay was initially optimized using reference gDNA samples from 10 bacterial species, including *C. jejuni*, *C. coli*, *Cl. perfringens*, *E. coli*, *Shigella* spp., *E. faecalis*, *E. faecium*, *L. monocytogenes* (both variant-1 and variant-2), *S. enterica*, and *S. aureus*. The resulting chromatogram (Figure 1) yielded the molecular mass of the SBE products for each designed target as expected, suggesting that the assay functioned effectively. All reactions with bacterial gDNA also demonstrated a mass spectrum of Bac16 1–1 SBE product (5816.80 Da). The complete chromatograms fully displayed the specific molecular mass of EPs and SBE products (Supplementary Figure S1). The negative control without any DNA template yielded only the peaks corresponding to the molecular mass of EPs without any of the SBE products or unexpected peaks, indicating a high specificity of the designed primers without any cross-reactivity resulting from the mixed primers.

When a non-targeted bacterium, *L. marthii*, was used as a template, the expected universal SBE product for bacteria was obtained, indicating suitability of the Bac16 1–1 primer set as an internal control to confirm the successful occurrence of all reactions and the validity of the results. Furthermore, when gDNA samples of non-bacterial samples, *C. fasciculata*, *L. martiniquensis*, SARS-CoV-2, human, and plant, were used as templates in the assay, the results showed no SBE products present in the reactions (Supplementary Figure S2).

**Figure 2:**
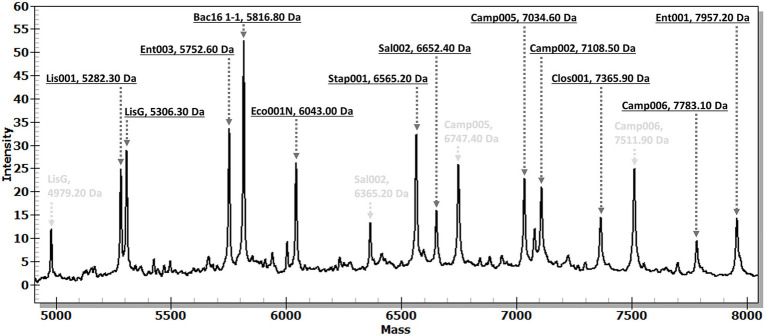
A chromatogram demonstrates the distinctive SBE products for bacterial targets within a mixed-species sample. The study used a mixed gDNA sample comprising 9 targeted species (10 bacterial strains), each at an equal concentration of 1 ng/μL. The black letters denote the molecular mass of SBE products associated with bacterial targets, while the gray letters indicate the remaining molecular mass of extension primers. Bac16 1–1 for universal bacteria, Camp002 for *C. jejuni*, Camp005 and Camp006 for *C. coli*, Clos001 for *Cl. perfringens*, Eco001N for *E. coli*, Ent001 for *E. faecium*, Ent003 for *E. faecalis*, Lis001 and LisG for *L. monocytogenes* variant-1 and variant-2, respectively. Sal002 for *Salmonella* spp., and Stap001 for *S. aureus* detection. The x-axis represents molecular mass (Da), and the y-axis represents peak intensity.

The evaluation of the developed MassARRAY-based assay’s reaction using a mixture of gDNA from all nine targeted species revealed the capability of a single MassARRAY-based assay to simultaneously detect all targeted species. The distinct molecular mass of the SBE products were clearly observed in the lowest concentration of gDNA as 1 ng/μL (Figure 2). When the mixture of gDNA from targeted bacteria and non-targeted bacteria (e.g., *E. coli*, *E. faecium*, *Salmonella* spp., *S. agalactiae*, *S. suis*, *C. fasciculata*, *L. martiniquensis*) was used as DNA templates, the complete chromatograms clearly displayed the specific molecular mass of specific SBE products of targeted bacterial species (data not shown). These results suggested the precision of the developed MassARRAY-based assay in accurately identifying the targeted bacteria species in a single run.

The developed MassARRAY-based assay underwent validation using gDNA samples obtained from pure cultures of well-identified bacterial species (*n* = 85; Supplementary Table S2). The MassARRAY results demonstrated 100% correlation between the molecular mass of SBE products and all bacterial species, with no occurrences of false-negative or false-positive results (Supplementary Table S3), highlighting the high accuracy of the assay. When non-targeted bacteria, including *C. lari*, *K. pneumoniae*, *L. innocua*, *S. agalactiae*, *S. suis*, and *V. parahaemolyticus*, sourced from distinct origins were used as templates, the complete chromatograms demonstrated a mass spectrum of Bac16 1–1 SBE product (5816.80 Da), which was the expected universal SBE product for bacteria (data not shown), indicating the validity of the results.

### 3.3 Limit of detection of the developed MassARRAY-based assay

The limit of detection (LOD) for the MassARRAY-based assay was assessed using serially diluted bacterial cell suspension, from which the gDNA was extracted using QuickExtract DNA Extraction Solution, as described in Section 2.7. The results revealed that the developed method achieved the lowest LOD in the reaction for *Cl. perfringens* at 357 ± 101 cells and *E. coli/Shigella* spp. at 445 ± 56 cells. The detection limit for *C. jejuni* (2,920 ± 651 cells) was comparable to that of *Salmonella* spp. (5,040 ± 825 cells). The LOD value for *E. faecalis* and *L. monocytogenes* was 36,000 ± 13,294 cells and 90,500 ± 48,790 cells, respectively. The highest LOD value was observed for *S. aureus* (282,000 ± 79,196 cells), *C. coli* (244,000 ± 10,392 cells), and *E. faecium* (221,000 ± 26,870 cells) (Table 2).

**Table 2:** Limit of detection of the MassARRAY-based assay.

Bacteria	Bacterial cells (mean ± SD)^*^
*Campylobacter coli*	244,000 ± 10,392
*Campylobacter jejuni*	2,920 ± 651
*Clostridium perfringens*	357 ± 101
*E. coli/Shigella* spp.	445 ± 56
*Enterococcus faecalis*	36,000 ± 13,294
*Enterococcus faecium*	221,000 ± 26,870
*Listeria monocytogenes*	90,500 ± 48,790
*Salmonella* spp.	5,040 ± 825
*Staphylococcus aureus*	282,000 ± 79,196

^*^The LOD values were expressed in terms of bacterial cells per assay reaction.

### 3.4 Validation of MassARRAY-based assay for bacterial identification in field samples

The performance of the developed MassARRAY-based assay in detecting targeted bacteria in livestock products was assessed using 103 meat samples collected from various sources. The meat samples underwent bacterial culture processing, which involved culture transport medium, selective plates, and enrichment plates. The assay validation was conducted across all stages of sample processing. The results obtained from the developed assay were subsequently compared to analyses conducted at the BQCLP laboratory. This comparison ensured the accuracy and reliability of the developed MassARRAY-based assay for bacterial identification in real-world application. Out of the 103 tested samples, 12 samples were positive for bacterial contamination, while the remaining samples (*n* = 91) tested negative for targeted bacterial species. These results were consistent with those obtained from bacterial culture-based method routinely conducted by the BQCLP laboratory. Notably, the negative control reaction without DNA template exhibited no SBE products, confirming the absence of contamination. All samples tested positive for Bac16 1–1, confirming the presence of bacteria in all field samples as expected. Furthermore, the specific molecular masses of the SBE products observed in the twelve positive samples matched the respective targeted bacteria, confirming the presence of specific bacterial species. The result demonstrated a 100% correlation between the data obtained from the standard bacterial-culture methods and the MassARRAY-based assay (Table 3). These findings indicated a high level of accuracy and reliability of the developed MassARRAY-based assay in bacterial identification, suggesting its potential for application within real-world field samples.

**Table 3:** Comparison of bacterial identification in field samples using the MassARRAY-based assay and bacterial culture-based method.

Sample no.	Sample ID	Bacterial culture-based method					MassARRAY-based assay				
		Bacteria	*E. coli/Shigella* spp.	*Enterococcus* spp.	*Salmonella* spp.	*S. aureus*	Bacteria	*E. coli/Shigella* spp.	*Enterococcus* spp.	*Salmonella* spp.	*S. aureus*
1	4627	P	N	N	N	N	P	N	N	N	N
2	4628	P	N	N	N	N	P	N	N	N	N
3	4646	P	P	N	N	N	P	P	N	N	N
4	5543	P	P	N	N	P	P	P	N	N	P
5	5545	P	N	N	N	N	P	N	N	N	N
6	5546	P	N	N	N	N	P	N	N	N	N
7	5555	P	N	N	N	N	P	N	N	N	N
8	5556	P	P	P	N	N	P	P	P^*^	N	N
9	5571	P	P	N	N	N	P	P	N	N	N
10	38532	P	P	P	P	N	P	P	P^*^	P	N
11	38633	P	P	P	P	P	P	P	P^*^	P	P
12	38809	P	P	P	N	N	P	P	P^*^	N	N

N, negative result; P, positive result.*The method specifically identified the species as *E. faecalis*.

## 4 Discussion

Given the ongoing threat of foodborne pathogens to global human health, several methods have been devised for detecting bacterial foodborne pathogens. While culture-based bacterial identification, considered the gold standard method, is widely employed in microbiology laboratories, it is recognized for its complexity, time-consuming procedures, and labor-intensive requirements (Nemati et al., 2016). An additional noteworthy technology in this field is MALDI-TOF MS (Khater et al., 2021). This advanced and rapid technique offers an alternative for bacterial identification. However, a limitation of this method is its reliance on reference data derived from specific protein fingerprints (Damodaran et al., 2007).

To address the constraint inherent in culture-based bacterial identification, nucleic acid detection-based approaches have undergone extensive expansion and widespread adoption (Shen et al., 2020; Bhattacharjee et al., 2021). Among these approaches, the combination of multiplex PCR with MALDI-TOF MS, referred to as MassARRAY technology, emerges as particularly advantageous. The unique feature of this technology enables the analysis of targeted genes based on molecular masses, eliminating the need for a reference genome database during data analysis and interpretation process. The MassARRAY technology is particularly well-suited for genotyping applications (Peng et al., 2013; Ellis and Ong, 2017; Hernandez et al., 2021). Moreover, numerous studies have demonstrated the versatility of this technology in successfully identifying pathogens across diverse microbial targets and disease contexts. For instance, Zhang et al. (2018) developed a method for the simultaneous detection of key bacterial pathogens related to pneumonia and meningitis using multiplex PCR coupled with mass spectrometry. This study has underscored the capability of MassARRAY technology in microbial detection, particularly in the context of infectious diseases. Additionally, Zhao et al. (2021) demonstrated the effectiveness of MassARRAY technology in detecting 27 respiratory pathogens, including both bacteria and viruses. In addition, successful applications of MassARRAY for pathogen identification in microbial detection have been reported (Syrmis et al., 2011; Trembizki et al., 2014; Yang et al., 2023).

While sharing similarities with other nucleic acid-based methods, the MassARRAY outperforms conventional PCR-based techniques by offering a unique capability to accommodate a larger number of primers within a single multiplexing reaction. However, the challenges of MassARRAY associated with multiplex PCR reaction, such as primer competition for targeted sequences could lead to reduced assay sensitivity, and the formation of nonspecific products or cross-reactions. To address these challenges, this study selected highly conserved regions from the multiple sequence alignments of targeted DNAs. The effectiveness of this process was enhanced by utilizing a substantial number of complete DNA sequences of the targeted genes, available in the NCBI database, for the sequence alignment. The Assay Design 4.0 software was employed with high stringent parameters to alleviate concerns related to secondary structures, homodimers, and heterodimers. This study also included a Bac16 1–1 primer set, specifically targeting 16S ribosomal RNA of bacteria, in the multiplex primer set. This addition aimed to enhance the assay’s ability to detect various bacterial species while also serving as an internal control within the assay system.

In the MassARRAY-based assay, precise adjustment of extension primer (EP) concentrations is crucial (Millis, 2011). Improper EP concentration can result in very low-intensity primer peaks during the assay, potentially leading to systematic failures when applied to samples as part of a multiplex. The EP mix could be varied depending on the number of plex reactions being processed. In this study, a 2-tier method recommended for the adjustment of EP concentration (iPLEX^®^ Pro and Gold Reagents USER GUIDE) was employed to optimize the amount of individual EP for 12 plex reaction. Furthermore, the peak intensity of all EPs was thoroughly examined. In cases where outlier peaks were observed for specific EPs, we recalibrated them by incorporating additional amounts of the particular EPs into the primer mixture, thereby ensuring uniform balance of peak intensity across all EP primer peaks. These comprehensive procedures were instrumental in the successful execution of the genotyping assay using MassARRAY technology.

In this study, two sets of primers, including Camp005 and Camp006 for *C. coli*, were designed to enhance species-specific detection while minimizing the occurrence of false negative results. Additionally, during this study, a novel SNP was discovered in the targeted gene of *L. monocytogenes* strains isolated from samples in Thailand. Therefore, two EPs, Lis001 and LisG, were incorporated into the assay to broaden the assay capability to detect the wild type and the newly identified variant strain, respectively.

It is noteworthy that the MassARRAY assay developed in this study lacks the necessary discriminatory capability to distinguish between *E. coli* and *Shigella* spp. This limitation stems from their close genetic resemblance and the absence of specific gene targets customized for such differentiation. Although several studies have reported specific primers to distinguish *Shigella* spp. from *E. coli*, it is important to acknowledge that those primers are still limited to certain pathogenic strains of *E. coli* and *Shigella* spp. (Liu, 2011; Halimeh et al., 2021). Due to the remarkably close genetic resemblance between *E. coli* and *Shigella* spp., reliably distinguishing them using molecular-based detection methods is extremely challenging. By considering the potential risk to human health posed by contamination with either *E. coli* or *Shigella* spp. in food products, regardless of the strain’s pathogenicity, a set of primers, Eco001N was designed to target a highly conserved region of the specific gene found in both *E. coli* and *Shigella* spp., allowing the simultaneous detection of both species in the sample. In this study, both inactive *E. coli* and indistinct species of *E. coli*/*Shigella* spp. samples were tested. A specific Eco001N base calling indicated potential contamination with either *E. coli* or *Shigella* spp., or both, and was reported as *E. coli*/*Shigella* spp. This approach is valuable for screening sample quality and preventing food contamination, ultimately safeguarding human health.

The developed MassARRAY-based assay demonstrated excellent efficiency, exhibiting a 100% correlation with known bacterial samples, previously identified by standard bacterial-culture methods. Moreover, it exhibited the ability to simultaneously differentiate targeted pathogens in co-contaminated samples, indicating considerable sensitivity and specificity of the assay. However, the detection limit of bacterial species identification in the MassARRAY-based assay was significantly variable. This may be attributed to several factors, including the complexity of the primer mixture in a single multiplex PCR reaction, the efficiency of bacterial cell lysis, and the stability of gDNA samples.

Three distinct DNA extraction kits were employed for specific purposes in this study. The ZymoBIOMICS DNA Miniprep Kit was used to obtain highly pure genomic DNA, particularly suitable for primer specificity testing and method development experiments. Additionally, the MagPurix^®^ Bacterial DNA Extraction Kit (ZP02006) with its automated extraction system was chosen for real-world applications, especially when handling a large number of samples daily and requiring a rapid and straightforward DNA extraction process. To minimize potential loss of genomic DNA from bacterial crude cell extracts, a simple manual DNA extraction method using QuickExtract DNA Extraction Solution with a heat-lysis protocol was implemented for preparing gDNA used for the LOD determination experiment. These approaches were implemented to ensure comprehensive and reliable DNA isolation for the various aspects of the study.

It is crucial to recognize that the variability of real samples obtained from different sources and the presence of background bacterial contamination in all samples could present challenges in limit of detection (LOD) determination when using real samples. Therefore, in this study, bacterial cell culture was employed for LOD determination instead of utilizing real samples. Although these calculated LOD values may not entirely reflect the realities of routine work, they still provide valuable insights into the performance of the method. It is important to highlight that the LOD of the developed assay exhibited a highly variable range (mean ± SD) of 357 ± 101 to 282,000 ± 79,196 cells in the assay reaction. This variability may be attributed to the efficiency of heat-lysis protocol used for quick DNA extraction and potential matrix interference in the reaction. However, our findings were consistent with a multiplex PCR assay that successfully detected pathogenic bacteria in spiked milk samples at a concentration of 10^4^ CFU/mL (Ashraf et al., 2017). Notably, achieving a LOD of one cell is quite challenging in multiplexing reactions, representing a recognized limitation of this approach. Moreover, the variability of gene targets and the quality of the nucleic acids are critical factors for the development of nucleic acid detection assays.

The application of the developed MassARRAY-based assay to real samples demonstrated a 100% concordance with the results of the standard culture-based method for four pathogens: *E. coli*, *Enterococcus* spp., *Salmonella* spp., and *S. aureus*. In field sample analysis, the developed assay efficiently detected *Salmonella* spp. at the initial stage when Buffered Peptone Water (BPW) was used as a transfer medium. This highlights the practical applicability of the MassARRAY-based assay for bacterial detection in real-world scenarios. Additionally, the MassARRAY assay conferred additional benefits over the standard culture-based method by simultaneously identifying *Enterococcus* spp. to the species level (*E. faecium* or *E. faecalis*) and identifying more targeted pathogen species without the need for additional steps. However, it is crucial to emphasize that when applying the MassARRAY-based assay to real samples, limitations may arise due to the detection limit of the multiplexing assay and/or the restricted sample volume in the reaction. To potentially overcome these constraints, incorporating selective enrichment steps for bacterial culture or introducing DNA extraction and purification steps could enhance the assay sensitivity. Furthermore, as DNA degradation over time could present challenges in detection, it is essential to prioritize the use of freshly prepared samples for the test.

In summary, the developed MassARRAY-based assay demonstrated its high-throughput performance in simultaneously identifying targeted species in a single assay with high specificity. Its successful application to field samples positioned it as a compelling choice for the rapid detection of foodborne bacterial pathogens in real-world field settings. Furthermore, the cost effectiveness, time efficiency, and accuracy inherent to MassARRAY technology make it highly suitable for adoption in food industrial laboratories, particularly those engaged in quality control of food products.

## Data availability statement

The original contributions presented in the study are included in the article/Supplementary material, further inquiries can be directed to the corresponding authors.

## Author contributions

NS: Writing – review & editing, Writing – original draft, Validation, Methodology, Investigation, Formal analysis, Data curation. SJ: Writing – review & editing, Methodology, Investigation, Formal analysis, Data curation. PS: Writing – review & editing, Methodology. PH: Writing – review & editing, Software, Resources. PA: Writing – review & editing, Supervision, Software, Resources, Project administration. MT: Writing – review & editing, Software, Project administration, Data curation. PP: Writing – review & editing, Software, Project administration, Data curation. NC: Writing – review & editing, Supervision, Resources, Conceptualization. SW: Writing – review & editing, Resources, Methodology, Data curation. PK: Writing – review & editing, Resources. SY: Writing – original draft, Writing – review & editing, Visualization, Validation, Supervision, Software, Resources, Project administration, Methodology, Investigation, Funding acquisition, Formal analysis, Data curation, Conceptualization.

## References

[ref1] AlMutawa F. Cabrera A. Chen F. Delport J. (2023). Performance of MassARRAY system for the detection of SARS-CoV-2 compared to real-time PCR. Eur J Microbiol Immunol (BP). 13, 1–5. doi: 10.1556/1886.2023.00013, PMID: 37318958 PMC10351579

[ref2] Ashraf A. Imran M. Yaqub T. Tayyab M. Shehzad W. Thomson P. C. (2017). A novel multiplex PCR assay for simultaneous detection of nine clinically significant bacterial pathogens associated with bovine mastitis. Mol. Cell. Probes 33, 57–64. doi: 10.1016/j.mcp.2017.03.004, PMID: 28336361

[ref3] Bhattacharjee G. Gohil N. Lam N. L. Singh V. (2021). CRISPR-based diagnostics for detection of pathogens. Prog. Mol. Biol. Transl. Sci. 181, 45–57. doi: 10.1016/bs.pmbts.2021.01.013 34127201

[ref4] Damodaran S. Wood T. D. Nagarajan P. Rabin R. A. (2007). Evaluating peptide mass fingerprinting-based protein identification. Genomics Proteomics Bioinformatics 5, 152–157. doi: 10.1016/S1672-0229(08)60002-9 18267296 PMC5054195

[ref5] Ellis J. A. Ong B. (2017). The MassARRAY® system for targeted SNP genotyping. Methods Mol. Biol. 1492, 77–94. doi: 10.1007/978-1-4939-6442-0_5, PMID: 27822857

[ref6] Gabriel S. Ziaugra L. Tabbaa D. (2009). SNP genotyping using the sequenom MassARRAY iPLEX platform. Curr Protoc Hum Genet 2:12. doi: 10.1002/0471142905.hg0212s60, PMID: 19170031

[ref7] Garzarelli V. Chiriacò M. S. Cereda M. Autuori I. Ferrara F. (2022). Miniaturized real-time PCR systems for SARS-CoV-2 detection at the point-of-care. Clin. Chim. Acta 536, 104–111. doi: 10.1016/j.cca.2022.09.014, PMID: 36126763 PMC9482443

[ref8] Halimeh F. B. Rafei R. Osman M. Kassem I. I. Diene S. M. Dabboussi F.. (2021). Historical, current, and emerging tools for identification and serotyping of Shigella. Braz. J. Microbiol. 52, 2043–2055. doi: 10.1007/s42770-021-00573-5, PMID: 34524650 PMC8441030

[ref9] Hernandez M. M. Banu R. Shrestha P. Patel A. Chen F. Cao L.. (2021). RT-PCR/MALDI-TOF mass spectrometry-based detection of SARS-CoV-2 in saliva specimens. J. Med. Virol. 93, 5481–5486. doi: 10.1002/jmv.27069, PMID: 33963565 PMC8242556

[ref10] Jaffee S. Henson S. Unnevehr L. Grace D. Cassou E. (2019). The Safe Food Imperative: Accelerating Progress in Low- and Middle-Income Countries. Washington, DC: World Bank.

[ref11] Kabiraz M. P. Majumdar P. R. Mahmud M. M. C. Bhowmik S. Ali A. (2023). Conventional and advanced detection techniques of foodborne pathogens: a comprehensive review. Heliyon 9:e15482. doi: 10.1016/j.heliyon.2023.e15482, PMID: 37151686 PMC10161726

[ref12] Khater D. F. Lela R. A. El-Diasty M. Moustafa S. A. Wareth G. (2021). Detection of harmful foodborne pathogens in food samples at the points of sale by MALDT-TOF MS in Egypt. BMC. Res. Notes 14:112. doi: 10.1186/s13104-021-05533-8, PMID: 33757586 PMC7988902

[ref13] Law J. W. Ab Mutalib N. S. Chan K. G. Lee L. H. (2015). Rapid methods for the detection of foodborne bacterial pathogens: principles, applications, advantages and limitations. Front. Microbiol. 5:770. doi: 10.3389/fmicb.2014.00770 25628612 PMC4290631

[ref14] Liu D. (2011). Molecular Detection of Human Bacterial Pathogens. 1st Edn. Boca Raton, Florida, United States: CRC Press.

[ref15] Millis M. P. (2011). “Medium-throughput SNP genotyping using mass spectrometry: multiplex SNP genotyping using the iPLEX® gold assay” in Disease Gene Identification. Methods in Molecular Biology. ed. DiStefano J., vol. 700 (Totowa, NJ: Humana Press). 10.1007/978-1-61737-954-3_5 21204027

[ref16] Min K. W. Kim W. S. Jang S. J. Choi Y. D. Chang S. Jung S. H.. (2016). MassARRAY, pyrosequencing, and PNA clamping for EGFR mutation detection in lung cancer tissue and cytological samples: a multicenter study. J. Cancer Res. Clin. Oncol. 142, 2209–2216. doi: 10.1007/s00432-016-2211-7, PMID: 27535566 PMC11819073

[ref17] National Antimicrobial Resistant Surveillance Center, Thailand. (2023). Thailand’s Integrated Antimicrobial Resistance Surveillance with One Health Approach Guideline. Available at: http://narst.dmsc.moph.go.th/ (Accessed May 15, 2024).

[ref18] Nemati M. Hamidi A. Maleki Dizaj S. Javaherzadeh V. Lotfipour F. (2016). An overview on novel microbial determination methods in pharmaceutical and food quality control. Adv Pharm Bull. 6, 301–308. doi: 10.15171/apb.2016.042, PMID: 27766214 PMC5071793

[ref19] Peng J. Yang F. Xiong Z. Guo J. Du J. Hu Y.. (2013). Sensitive and rapid detection of viruses associated with hand foot and mouth disease using multiplexed MALDI-TOF analysis. J Clin Viro. 56, 170–174. doi: 10.1016/j.jcv.2012.10.020 23194776

[ref20] Shen M. Zhou Y. Ye J. Abdullah Al-Maskri A. A. Kang Y. Zeng S.. (2020). Recent advances and perspectives of nucleic acid detection for coronavirus. J Pharm Anal. 10, 97–101. doi: 10.1016/j.jpha.2020.02.010, PMID: 32292623 PMC7102540

[ref21] Syrmis M. W. Moser R. J. Whiley D. M. Vaska V. Coombs G. W. Nissen M. D.. (2011). Comparison of a multiplexed MassARRAY system with real-time allele-specific PCR technology for genotyping of methicillin-resistant *Staphylococcus aureus*. Clin. Microbiol. Infect. 17, 1804–1810. doi: 10.1111/j.1469-0691.2011.03521.x, PMID: 21595795

[ref22] Tan M. Liao C. Liang L. Yi X. Zhou Z. Wei G. (2022). Recent advances in recombinase polymerase amplification: principle, advantages, disadvantages and applications. Front. Cell. Infect. Microbiol. 12:1019071. doi: 10.3389/fcimb.2022.1019071, PMID: 36519130 PMC9742450

[ref23] The United States Food and Drug Administration. (2024). Bacteriological Analytical Manual (BAM). Available at: https://www.fda.gov/food/science-research-food/laboratory-methods-food (Accessed May 15, 2024).

[ref24] Thompson J. D. Higgins D. G. Gibson T. J. (1994). CLUSTAL W: improving the sensitivity of progressive multiple sequence alignment through sequence weighting, position-specific gap penalties and weight matrix choice. Nucleic Acids Res. 22, 4673–4680. doi: 10.1093/nar/22.22.4673, PMID: 7984417 PMC308517

[ref1002] Trembizki E. Smith H. Lahra M. M. Chen M. Donovan B. Fairley C. K.. (2014). High-throughput informative single nucleotide polymorphism-based typing of *Neisseria gonorrhoeae* using the Sequenom MassARRAY iPLEX platform. J Antimicrob Chemother. 69, 1526–32. doi: 10.1093/jac/dkt544, PMID: 24468863

[ref25] Wacharapluesadee S. Hirunpatrawong P. Petcharat S. Torvorapanit P. Jitsatja A. Thippamom N.. (2023). Simultaneous detection of omicron and other SARS-CoV-2 variants by multiplex PCR MassARRAY technology. Sci. Rep. 13:2089. doi: 10.1038/s41598-023-28715-9, PMID: 36747014 PMC9900542

[ref26] Wang K. Wang G. Huang S. Luo A. Jing X. Li G.. (2019). Association between TIMP-2 gene polymorphism and breast cancer in Han Chinese women. BMC Cancer 19:446. doi: 10.1186/s12885-019-5655-8, PMID: 31088428 PMC6518501

[ref27] World Health Organization (2022). Food Safety. Available at: https://www.who.int/news-room/fact-sheets/detail/food-safety (Accessed May 15, 2024).

[ref1003] Yang H. Li A. Dang L. Kang T. Ren F. Ma J.. (2023). A rapid, accurate, and low-cost method for detecting *Mycobacterium tuberculosis* and its drug-resistant genes in pulmonary tuberculosis: Applications of MassARRAY DNA mass spectrometry. Front Microbiol. 14:1093745. doi: 10.3389/fmicb.2023.1093745, PMID: 36910195 PMC9996023

[ref1004] Zhang C. Xiu L. Xiao Y. Xie Z. Ren L. Peng J.. (2018). Simultaneous detection of key bacterial pathogens related to pneumonia and meningitis using multiplex PCR coupled with mass spectrometry. Front Cell Infect Microbiol. 8:107. doi: 10.3389/fcimb.2018.00107, PMID: 29675400 PMC5895723

[ref1005] Zhao H. Yang Y. Lyu J. Ren X. Cheng W. (2021). Development and application of a method to detect 27 respiratory pathogens using multiplex RT-PCR combined with MassARRAY technology. BMC Infect Dis. 21:870. doi: 10.1186/s12879-021-06404-0, PMID: 34433411 PMC8385475

[ref28] Zowawi H. M. Syrmis M. W. Kidd T. J. Balkhy H. H. Walsh T. R. Al Johani S. M.. (2018). Identification of carbapenem-resistant *Pseudomonas aeruginosa* in selected hospitals of the Gulf cooperation council states: dominance of high-risk clones in the region. J. Med. Microbiol. 67, 846–853. doi: 10.1099/jmm.0.000730, PMID: 29664716

