# Supplementary figures and images for: MassARRAY: a high-throughput solution for rapid detection of foodborne pathogens in real-world settings
Source: Front Microbiol. 2024 Jun 25;15:1403579. doi: 10.3389/fmicb.2024.1403579 (PMC11232118; doi:10.3389/fmicb.2024.1403579)

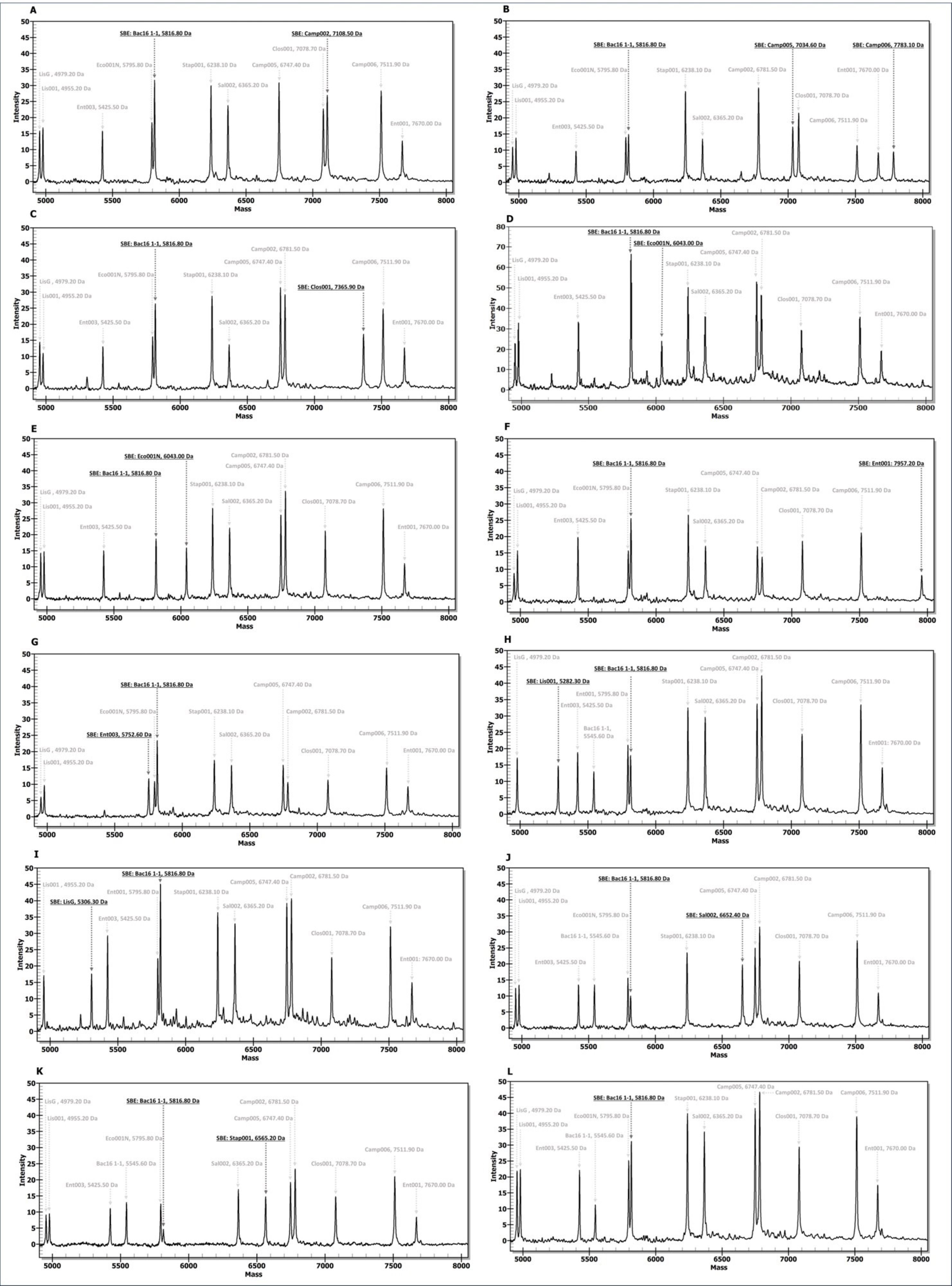

Supplement: Supplementary file 1 [file Image_1.jpg]

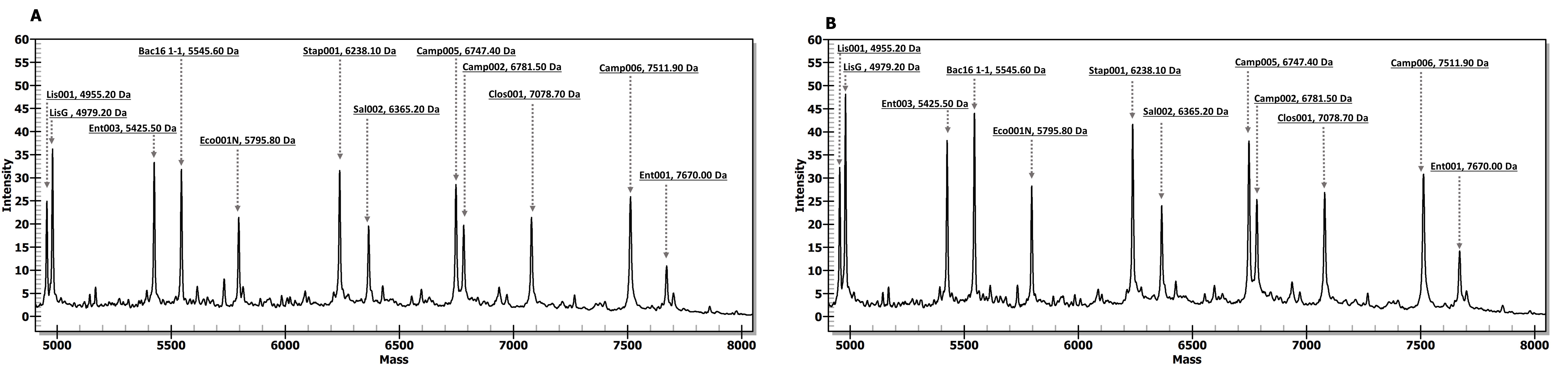

Supplement: Supplementary file 2 [file Image_2.jpg]
