# Supplementary material for: MassARRAY: a high-throughput solution for rapid detection of foodborne pathogens in real-world settings
Source: Front Microbiol. 2024 Jun 25;15:1403579. doi: 10.3389/fmicb.2024.1403579 (PMC11232118; doi:10.3389/fmicb.2024.1403579)
Supplement: Supplementary file 3 [file Data_Sheet_2.docx]

Supplementary Material

**MassARRAY: A High-Throughput Solution for Rapid Detection of Foodborne Pathogens in Real-World Settings**

**Namfon Suebwongsa*, Surasak Jiemsup, Pannita Santiyanont, Piyapha Hirunpatrawong, Pornsiri Aswapairin, Monthathip Thongkum, Prakaymars Panumars, Nipa Chokesajjawatee, Supaporn Wongsrichai, Pichet Koompa, and Suganya Yongkiettrakul***

***Correspondence:** Namfon Suebwongsa: [namfon.suebwongsa@gmail.com](mailto:namfon.suebwongsa@gmail.com), Suganya Yongkiettrakul: [suganya.yon@biotec.or.th](mailto:suganya.yon@biotec.or.th)

# Supplementary Tables

**Supplementary Table 1.** Genomic DNA samples used as reference DNA templates in this study.

|  | **No.** | **Samples (gDNA)** | **Strains** | **Sources** |
| --- | --- | --- | --- | --- |
| Bacteria | 1 | *Campylobacter coli* | JV20 | ATCC |
|  | 2 | *C. jejuni* | D3071 | ATCC |
|  | 3 | *Clostridium perfringens* | WAL-14572 | ATCC |
|  | 4 | *Escherichia coli* | B171 | ATCC |
|  | 5 | *Enterococcus faecalis* | TX0104 | ATCC |
|  | 6 | *E. faecalis* | TUSoD Ef11 | ATCC |
|  | 7 | *Enterococcus faecium* | TX0133a04 | ATCC |
|  | 8 | *Listeria marthii* | FSL S4-120 | ATCC |
|  | 9 | *L. monocytogenes* | F6900 | ATCC |
|  | 10 | *L. monocytogenes* | Li21 | ATCC |
|  | 11 | *Salmonella enterica* | 15/5 | ATCC |
|  | 12 | *S. enterica* | LT2 | ATCC |
|  | 13 | *Shigella* spp. | NMICID504 | Human *^a^* |
|  | 14 | *Staphylococcus aureus* | CM05 | ATCC |
| Protozoa | 15 | *Crithidia fasciculata* | ND | Human *^b^* |
|  | 16 | *Leishmania martiniquensis* | ND | Human *^b^* |
| Virus | 17 | Severe acute respiratory  syndrome coronavirus 2 (SAR-CoV-2) | ND | Human *^b^* |
| Human | 18 | Human | No.4312660 | Applied Biosystems, California,  United States. |
| Plant | 19 | Plant | ND | Teak leaves *^c^* |

ATCC: American Type Culture Collection, ND: no data available, *^a^*: Faculty of Medicine Ramathibodi Hospital, Mahidol University, Thailand, *^b^*: Faculty of Medicine, Chulalongkorn University, Thailand., and *^c^*: Lifomics Co., Ltd., Thailand.

**Supplementary Table 2.** Bacterial strains were used in this study.

| **No.** | **Species** | **Strains ID** | **Source** |
| --- | --- | --- | --- |
| 1 | *Campylobacter coli* | S2-4 M8-4 | Food |
| 2 | *C. coli* | VA2 M1-1 | Food |
| 3 | *C. coli* | VA2 S2-4 | Environment |
| 4 | *C. jejuni* | ATCC33560 | Bovine |
| 5 | *C. jejuni* | S3-3 M13-1 | Chicken |
| 6 | *C. jejuni* | S3-3 M14-1 | Chicken |
| 7 | *C. jejuni* | S2-5 M11-1 | Food |
| 8 | *C. jejuni* | S2-5 M13-4 | Food |
| 9 | *C. jejuni* | S2-6 M7-1 | Chicken |
| 10 | *C. jejuni* | S2-6 S21-1 | Environment |
| 11 | *C. jejuni* | S2-6 W7-1 | Environment |
| 12 | *C. lari* | ATCC35223 | Human |
| 13 | *Clostridium bifermentans* | FBU0548 | Buffalo |
| 14 | *Cl. butyricum* | FBU0266 | Human |
| 15 | *Cl. butyricum* | FBU0267 | Human |
| 16 | *Cl. perfringens* | FBU0184 | Human |
| 17 | *Cl. perfringens* | FBU0241 | Human |
| 18 | *Cl. perfringens* | FBU0242 | Human |
| 19 | *Cl. perfringens* | FBU0960 | Chicken |
| 20 | *Cl. perfringens* | FBU1161 | Pig |
| 21 | *Enterococcus faecalis* | CGAMO01 | Chicken |
| 22 | *E. faecalis* | CGAMO02 | Chicken |
| 23 | *E. faecalis* | CGBMO01 | Chicken |
| 24 | *E. faecalis* | CGBMO07 | Chicken |
| 25 | *E. faecalis* | GMBMO03 | Goat |
| 26 | *E. faecalis* | HBBMO06 | Human |
| 27 | *E. faecalis* | HBBMO23 | Human |
| 28 | *E. faecalis* | JCM5803 | ND |
| 29 | *E. faecalis* | PGAMO46 | Pig |
| 30 | *E. faecalis* | PGAMO21 | Pig |
| 31 | *E. faecalis* | PGAMO85 | Pig |
| 32 | *E. faecium* | GMBMO02 | Goat |
| 33 | *E. faecium* | GMBMO05 | Goat |
| 34 | *E. faecium* | GMCMO08 | Goat |
| 35 | *E. faecium* | GMCMO07 | Goat |
| 36 | *E. faecium* | JCM 5804 | ND |
| 37 | *Escherichia coli* | ATCC25404 | ND |
| 38 | *E. coli* | ATCC25922 | Human |
| 39 | *E. coli* (inactive strain) | FBU112 | Human |
| 40 | *E. coli* (inactive strain) | FBU1084 | Pig |
| 41 | *E. coli/ Shigella spp.* | BFCMO04 | Animal feces |
| 42 | *E. coli/ Shigella spp.* | HFBMO13 | Human |
| 43 | *E. coli/ Shigella spp.* | HFCMO06 | Human |
| 44 | *E. coli/ Shigella spp.* | PFBMO01 | Animal feces |
| 45 | *Klebsiella pneumoniae* | HBAMO11 | Human |
| 46 | *Listeria inocua* | ATCC33090 | Cow |
| 47 | *L. inocua* | ATCC51742 | Food |
| 48 | *L. monocytogenes* | ATCC19112 | Human |
| 49 | *L. monocytogenes* | ATCC19115 | Human |
| 50 | *L. monocytogenes* | F6900 | Human |
| 51 | *L. monocytogenes* | MTS016 | Food |
| 52 | *L. monocytogenes* | MTS017 | Food |
| 53 | *L. monocytogenes* | MTS018 | Food |
| 54 | *L. monocytogenes* | MTS019 | Food |
| 55 | *L. monocytogenes* | 10403S | Human |
| 56 | *Salmonella* Agona | 1499 | Chicken |
| 57 | *Salmonella* Albany | 1579 | Chicken |
| 58 | *Salmonella* Altona | 1669 | Chicken |
| 59 | *Salmonella* Choleraesuis | ATCC10708 | ND |
| 60 | *Salmonella* Corvallis | IV-1010 | Chicken |
| 61 | *Salmonella* Derby | 3484 | Chicken |
| 62 | *Salmonella* Enteritidis | 091 | Environment |
| 63 | *Salmonella* Essen | ATCC6961 | ND |
| 64 | *Salmonella* Give | 1589 | Chicken |
| 65 | *Salmonella* Hadar | DMST32761 | ND |
| 66 | *Salmonella* Infantis | DMST49110 | ND |
| 67 | *Salmonella* Orion | 3806 | Chicken |
| 68 | *Salmonella* Paratyphi B variety java | 3489 | Chicken |
| 69 | *Salmonella* Stanley | 3536 | Chicken |
| 70 | *Salmonella* Typhimurium | ATCC13311 | Human |
| 71 | *Salmonella* Typhimurium | 003 | Environment |
| 72 | *Salmonella* Virchow | 2969 | Chicken |
| 73 | *Salmonella* Weltevreden | 0380 | Environment |
| 74 | *Staphylococcus aureus* | ATCC14458 | Human |
| 75 | *S. aureus* | ATCC25923 | Human |
| 76 | *S. aureus* | SA14 | Food |
| 77 | *S. aureus* | SA33 | Food |
| 78 | *S. aureus* | SA63 | Food |
| 79 | *S. aureus* | SA101 | Food |
| 80 | *S. aureus* | SA127 | Food |
| 81 | *S. aureus* | SA129 | Food |
| 82 | *S. aureus* | SA182 | Food |
| 83 | *Streptococcus agalactiae* | IAPTSA001 | Fish |
| 84 | *S. suis* | IAPTSS191 | Pig |
| 85 | *Vibrio parahaemolyticus* | ATCC17802 | ND |

ND: No data available.

**Supplementary Table 3.** The identification of bacterial species using the MassARRAY-based assay.

| **No.** | **gDNA samples from pure colony species** | **Strains ID** | **Base calling** | | | | | | | | | | | | **MassARRAY interpretation** |
| --- | --- | --- | --- | --- | --- | --- | --- | --- | --- | --- | --- | --- | --- | --- | --- |
|  |  |  | **Bac16**  **1-1** | **Camp002** | **Camp005** | **Camp006** | **Clos**  **001** | **Eco**  **001N** | **Ent**  **001** | **Ent**  **003** | **Lis**  **001** | **LisG** | **Sal**  **002** | **Stap**  **001** |  |
| 1 | *Campylobacter coli* | S2-4 M8-4 | + | - | + | + | - | - | - | - | - | - | - | - | *C. coli* |
| 2 | *C. coli* | VA2 M1-1 | + | - | + | + | - | - | - | - | - | - | - | - | *C. coli* |
| 3 | *C. coli* | VA2 S2-4 | + | - | + | + | - | - | - | - | - | - | - | - | *C. coli* |
| 4 | *C. jejuni* | ATCC  33560 | + | + | - | - | - | - | - | - | - | - | - | - | *C. jejuni* |
| 5 | *C. jejuni* | S3-3 M13-1 | + | + | - | - | - | - | - | - | - | - | - | - | *C. jejuni* |
| 6 | *C. jejuni* | S3-3 M14-1 | + | + | - | - | - | - | - | - | - | - | - | - | *C. jejuni* |
| 7 | *C. jejuni* | S2-5 M11-1 | + | + | - | - | - | - | - | - | - | - | - | - | *C. jejuni* |
| 8 | *C. jejuni* | S2-5 M13-4 | + | + | - | - | - | - | - | - | - | - | - | - | *C. jejuni* |
| 9 | *C. jejuni* | S2-6 M7-1 | + | + | - | - | - | - | - | - | - | - | - | - | *C. jejuni* |
| 10 | *C. jejuni* | S2-6 S21-1 | + | + | - | - | - | - | - | - | - | - | - | - | *C. jejuni* |
| 11 | *C. jejuni* | S2-6 W7-1 | + | + | - | - | - | - | - | - | - | - | - | - | *C. jejuni* |
| 12 | *Clostridium perfringens* | FBU  0184 | + | - | - | - | + | - | - | - | - | - | - | - | *Cl. perfringens* |
| 13 | *Cl. perfringens* | FBU  0241 | + | - | - | - | + | - | - | - | - | - | - | - | *Cl. perfringens* |
| 14 | *Cl. perfringens* | FBU  0242 | + | - | - | - | + | - | - | - | - | - | - | - | *Cl. perfringens* |
| 15 | *Cl. perfringens* | FBU  0960 | + | - | - | - | + | - | - | - | - | - | - | - | *Cl. perfringens* |
| 16 | *Cl. perfringens* | FBU  1161 | + | - | - | - | + | - | - | - | - | - | - | - | *Cl. perfringens* |
| 17 | *Escherichia coli* | ATCC  25404 | + | - | - | - | - | + | - | - | - | - | - | - | *E.coli* |
| 18 | *E. coli* | ATCC  25922 | + | - | - | - | - | + | - | - | - | - | - | - | *E. coli* |
| 19 | *E. coli*  (inactive strain) | FBU  112 | + | - | - | - | - | + | - | - | - | - | - | - | *E. coli* |
| 20 | *E. coli*  (inactive strain) | FBU  1084 | + | - | - | - | - | + | - | - | - | - | - | - | *E. coli* |
| 21 | *E. coli/ Shigella spp.* | BFCMO04 | + | - | - | - | - | + | - | - | - | - | - | - | *E. coli/ Shigella spp.* |
| 22 | *E. coli/ Shigella spp.* | HFAMO13 | + | - | - | - | - | + | - | - | - | - | - | - | *E. coli/ Shigella spp.* |
| 23 | *E. coli/ Shigella spp.* | HFCMO06 | + | - | - | - | - | + | - | - | - | - | - | - | *E. coli/ Shigella spp.* |
| 24 | *E. coli/ Shigella spp.* | PFBMO01 | + | - | - | - | - | + | - | - | - | - | - | - | *E. coli/ Shigella spp.* |
| 25 | *Enterococcus faecalis* | CGAMO01 | + | - | - | - | - | - | - | + | - | - | - | - | *E. faecalis* |
| 26 | *E. faecalis* | CGAMO02 | + | - | - | - | - | - | - | + | - | - | - | - | *E. faecalis* |
| 27 | *E. faecalis* | CGBMO01 | + | - | - | - | - | - | - | + | - | - | - | - | *E. faecalis* |
| 28 | *E. faecalis* | CGBMO07 | + | - | - | - | - | - | - | + | - | - | - | - | *E. faecalis* |
| 29 | *E. faecalis* | GMBMO03 | + | - | - | - | - | - | - | + | - | - | - | - | *E. faecalis* |
| 30 | *E. faecalis* | HBBMO06 | + | - | - | - | - | - | - | + | - | - | - | - | *E. faecalis* |
| 31 | *E. faecalis* | HBBMO23 | + | - | - | - | - | - | - | + | - | - | - | - | *E. faecalis* |
| 32 | *E. faecalis* | JCM5803 | + | - | - | - | - | - | - | + | - | - | - | - | *E. faecalis* |
| 33 | *E. faecalis* | PGAMO46 | + | - | - | - | - | - | - | + | - | - | - | - | *E. faecalis* |
| 34 | *E. faecalis* | PGAMO21 | + | - | - | - | - | - | - | + | - | - | - | - | *E. faecalis* |
| 35 | *E. faecalis* | PGAMO85 | + | - | - | - | - | - | - | + | - | - | - | - | *E. faecalis* |
| 36 | *E. faecium* | GMBMO02 | + | - | - | - | - | - | + | - | - | - | - | - | *E. faecium* |
| 37 | *E. faecium* | GMBMO05 | + | - | - | - | - | - | + | - | - | - | - | - | *E. faecium* |
| 38 | *E. faecium* | GMCMO08 | + | - | - | - | - | - | + | - | - | - | - | - | *E. faecium* |
| 39 | *E. faecium* | GMCMO07 | + | - | - | - | - | - | + | - | - | - | - | - | *E. faecium* |
| 40 | *E. faecium* | JCM 5804 | + | - | - | - | - | - | + | - | - | - | - | - | *E. faecium* |
| 41 | *L. monocytogenes* | ATCC  19112 | + | - | - | - | - | - | - | - | + | - | - | - | *L. monocytogenes*  (variant-1) |
| 42 | *L. monocytogenes* | ATCC  19115 | + | - | - | - | - | - | - | - | + | - | - | - | *L. monocytogenes*  (variant-1) |
| 43 | *L. monocytogenes* | F6900 | + | - | - | - | - | - | - | - | + | - | - | - | *L. monocytogenes*  (variant-1) |
| 44 | *L. monocytogenes* | 10403S | + | - | - | - | - | - | - | - | + | - | - | - | *L. monocytogenes*  (variant-1) |
| 45 | *L. monocytogenes* | MTS  016 | + | - | - | - | - | - | - | - | - | + | - | - | *L. monocytogenes* (variant-2) |
| 46 | *L. monocytogenes* | MTS  017 | + | - | - | - | - | - | - | - | - | + | - | - | *L. monocytogenes*  (variant-2) |
| 47 | *L. monocytogenes* | MTS  018 | + | - | - | - | - | - | - | - | - | + | - | - | *L. monocytogenes*  (variant-2) |
| 48 | *L. monocytogenes* | MTS  019 | + | - | - | - | - | - | - | - | - | + | - | - | *L. monocytogenes*  (variant-2) |
| 49 | *Salmonella Agona* | 1499 | + | - | - | - | - | - | - | - | - | - | + | - | *Salmonella spp.* |
| 50 | *Salmonella Albany* | 1579 | + | - | - | - | - | - | - | - | - | - | + | - | *Salmonella spp.* |
| 51 | *Salmonella Altona* | 1669 | + | - | - | - | - | - | - | - | - | - | + | - | *Salmonella spp.* |
| 52 | *Salmonella Choleraesuis* | ATCC  10708 | + | - | - | - | - | - | - | - | - | - | + | - | *Salmonella spp.* |
| 53 | *Salmonella Corvallis* | IV-1010 | + | - | - | - | - | - | - | - | - | - | + | - | *Salmonella spp.* |
| 54 | *Salmonella Derby* | 3484 | + | - | - | - | - | - | - | - | - | - | + | - | *Salmonella spp.* |
| 55 | *Salmonella Enteritidis* | 091 | + | - | - | - | - | - | - | - | - | - | + | - | *Salmonella spp.* |
| 56 | *Salmonella Essen* | ATCC  6961 | + | - | - | - | - | - | - | - | - | - | + | - | *Salmonella spp.* |
| 57 | *Salmonella Give* | 1589 | + | - | - | - | - | - | - | - | - | - | + | - | *Salmonella spp.* |
| 58 | *Salmonella Hadar* | DMST  32761 | + | - | - | - | - | - | - | - | - | - | + | - | *Salmonella spp.* |
| 59 | *Salmonella Infantis* | DMST  49110 | + | - | - | - | - | - | - | - | - | - | + | - | *Salmonella spp.* |
| 60 | *Salmonella Orion* | 3806 | + | - | - | - | - | - | - | - | - | - | + | - | *Salmonella spp.* |
| 61 | *Salmonella Paratyphi B variety java* | 3489 | + | - | - | - | - | - | - | - | - | - | + | - | *Salmonella spp.* |
| 62 | *Salmonella Stanley* | 3536 | + | - | - | - | - | - | - | - | - | - | + | - | *Salmonella spp.* |
| 63 | *Salmonella Typhimurium* | ATCC  13311 | + | - | - | - | - | - | - | - | - | - | + | - | *Salmonella spp.* |
| 64 | *Salmonella Typhimurium* | 003 | + | - | - | - | - | - | - | - | - | - | + | - | *Salmonella spp.* |
| 65 | *Salmonella Virchow* | 2969 | + | - | - | - | - | - | - | - | - | - | + | - | *Salmonella spp.* |
| 66 | *Salmonella Weltevreden* | 380 | + | - | - | - | - | - | - | - | - | - | + | - | *Salmonella spp.* |
| 67 | *Staphylococcus aureus* | ATCC  14458 | + | - | - | - | - | - | - | - | - | - | - | + | *S. aureus* |
| 68 | *S. aureus* | ATCC  25923 | + | - | - | - | - | - | - | - | - | - | - | + | *S. aureus* |
| 69 | *S. aureus* | SA14 | + | - | - | - | - | - | - | - | - | - | - | + | *S. aureus* |
| 70 | *S. aureus* | SA33 | + | - | - | - | - | - | - | - | - | - | - | + | *S. aureus* |
| 71 | *S. aureus* | SA63 | + | - | - | - | - | - | - | - | - | - | - | + | *S. aureus* |
| 72 | *S. aureus* | SA101 | + | - | - | - | - | - | - | - | - | - | - | + | *S. aureus* |
| 73 | *S. aureus* | SA127 | + | - | - | - | - | - | - | - | - | - | - | + | *S. aureus* |
| 74 | *S. aureus* | SA129 | + | - | - | - | - | - | - | - | - | - | - | + | *S. aureus* |
| 75 | *S. aureus* | SA182 | + | - | - | - | - | - | - | - | - | - | - | + | *S. aureus* |
| 76 | *C. lari* | ATCC  35223 | + | - | - | - | - | - | - | - | - | - | - | - | *Bacteria* |
| 77 | *Cl. bifermentans* | FBU0548 | + | - | - | - | - | - | - | - | - | - | - | - | *Bacteria* |
| 78 | *Cl. butyricum* | FBU0266 | + | - | - | - | - | - | - | - | - | - | - | - | *Bacteria* |
| 79 | *Cl. butyricum* | FBU0267 | + | - | - | - | - | - | - | - | - | - | - | - | *Bacteria* |
| 80 | *K. pneumoniae* | HBAMO11 | + | - | - | - | - | - | - | - | - | - | - | - | *Bacteria* |
| 81 | *L. inocua* | ATCC  33090 | + | - | - | - | - | - | - | - | - | - | - | - | *Bacteria* |
| 82 | *L. inocua* | ATCC  51742 | + | - | - | - | - | - | - | - | - | - | - | - | *Bacteria* |
| 83 | *Streptococcus*  *agalactiae* | IAPT  SA001 | + | - | - | - | - | - | - | - | - | - | - | - | *Bacteria* |
| 84 | *S. suis* | IAPT  SS191 | + | - | - | - | - | - | - | - | - | - | - | - | *Bacteria* |
| 85 | *Vibrio parahaemolyticus* | ATCC  17802 | + | - | - | - | - | - | - | - | - | - | - | - | *Bacteria* |

+: Positive base calling, -: Negative base calling. The result was obtained from three independent experiments.

## Supplementary Figures


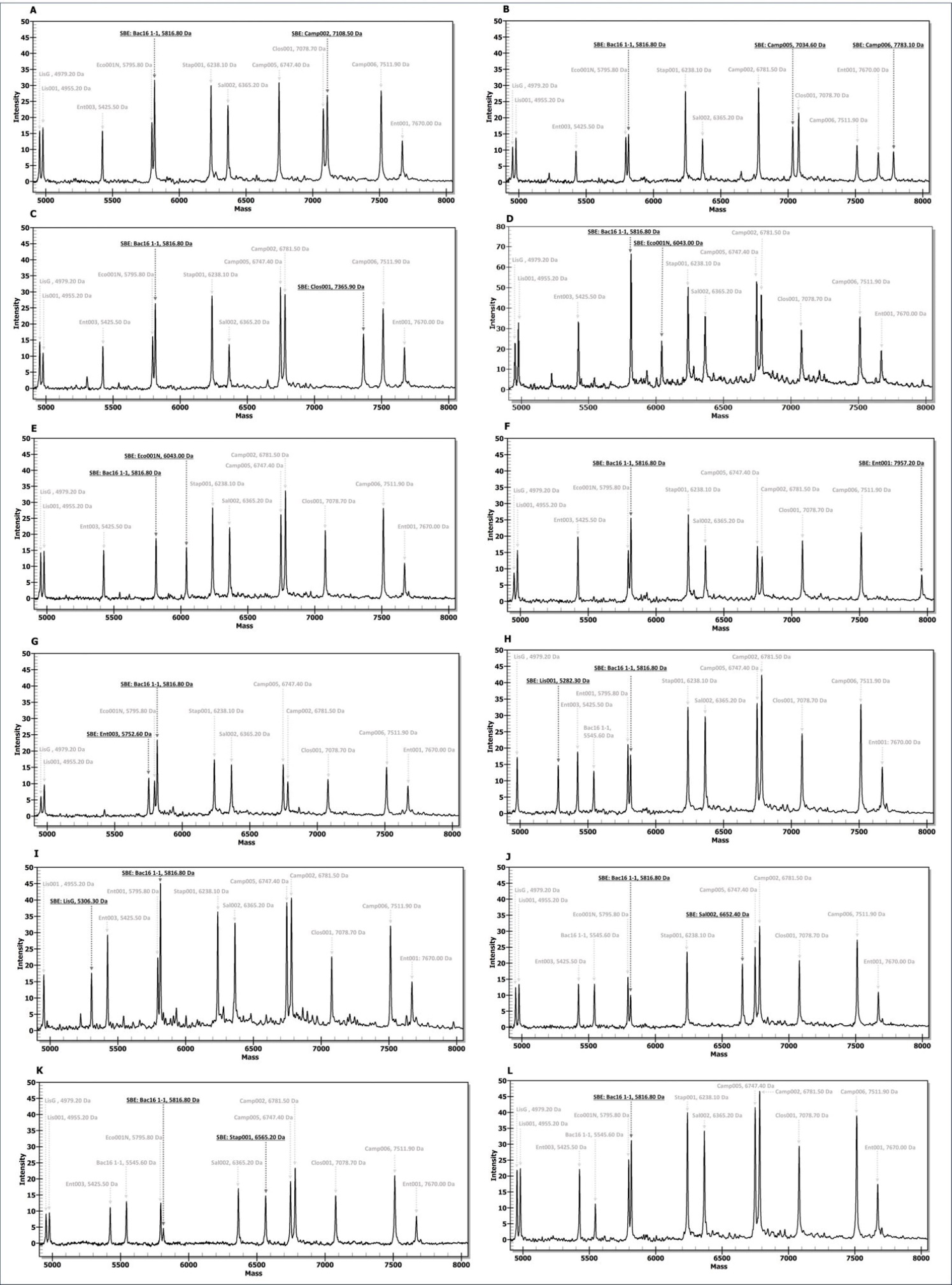


**Supplementary Figure 1.** The chromatograms displayed the specific molecular mass of specific products. The symbols including **(A)** represented the molecular mass of Bac16 1-1 (5816.80 Da) and Cmp002 (7108.50 Da) for *C. jejuni* detection, **(B)** represented the molecular mass of Bac16 1-1 (5816.80 Da), Cmp005 (7034.60 Da) and Cmp006 (7783.10 Da) for *C. coli*, **(C)** represented the molecular mass of Bac16 1-1 (5816.80 Da) and Clos001 (7365.90 Da) for *C. perfringens* detection, **(D)** represented the molecular mass of Bac16 1-1 (5816.80 Da) and Eco001N (6043.00 Da) for *E. coli* detection, **(E)** represented the molecular mass of Bac16 1-1 (5816.80 Da) and Eco001N (6043.00 Da) for *Shigella* spp. detection, **(F)** represented the molecular mass of Bac16 1-1 (5816.80 Da) and Ent001 (7957.20 Da) for *E. faecium* detection, **(G)** represented the molecular mass of Bac16 1-1 (5816.80 Da) and Ent003 (5752.60 Da) for *E. faecalis* detection, **(H)** represented the molecular mass of Bac16 1-1 (5816.80 Da) and Lis001 (5282.30 Da) for *L. monocytogenes* variant-1 detection, **(I)** represented the molecular mass of Bac16 1-1 (5816.80 Da) and LisG (5306.30 Da) for *L. monocytogenes* variant-2 detection, **(J)** represented the molecular mass of Bac16 1-1 (5816.80 Da) and Sal002 (6652.40 Da) for *Salmonella* spp. detection, and **(K)** represented the molecular mass of Bac16 1-1 (5816.80 Da) and Stap001 (6565.20 Da) for *S. aureus* detection. **(L)** represented the molecular mass of Bac16 1-1 (5816.80 Da) in the *L. marthii*. X-axis represents molecular mass (Da) y-axis represents peak intensity.

**
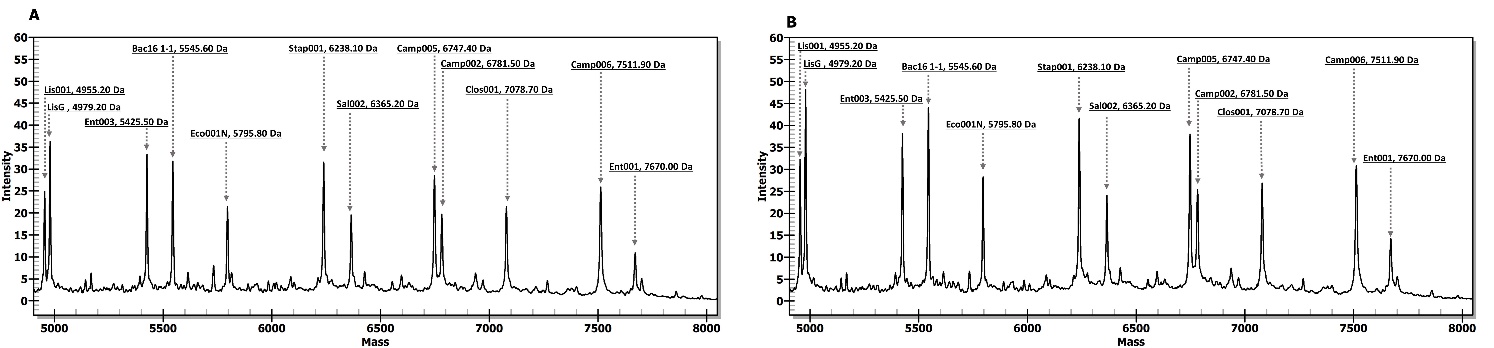
**

**Supplementary Figure 2.** The chromatograms representing negative results clearly illustrate specific molecular mass of the extension primers when human gDNA (A) and plant gDNA (B) were used as DNA templates in the MassARRAY-based assay. Similar chromatograms were observed for additional negative controls, including *Crithidia fasciculata*, *Leishmania martiniquensis*, and Severe Acute Respiratory Syndrome Coronavirus 2 (SARS-CoV-2). These chromatograms indicated consistent negative results across different non-targeted organisms, thus confirming the absence of cross-reaction in the MassARRAY-based assay system.
